# Sensitivity analysis of heat and mass transfer characteristics during forced‐air cooling process of peaches on different air‐inflow velocities

**DOI:** 10.1002/fsn3.1951

**Published:** 2020-10-20

**Authors:** Ying‐Min Chen, Hai‐Yan Song, Zhen‐Shi Chen, Rui Zhao, Qin Su, Peng‐Yong Jin, Yi‐Shu Sun, Hao Wang

**Affiliations:** ^1^ College of Agricultural Engineering Shanxi Agricultural University Taigu China; ^2^ Guangdong Provincial Key Laboratory of Optical Fiber Sensing and Communications Institute of Photonics Technology Jinan University Guangzhou China; ^3^ State Key Laboratory of Optoelectronic Materials and Technologies and School of Electronics and Information Technology Sun Yat‐Sen University Guangzhou China

**Keywords:** computational fluid dynamics, heat source term, forced‐air cooling, precooling performance, freshly harvested peaches

## Abstract

Peach is one of the most perishable fruits. During forced‐convection cooling, the heat sources (respiratory and evaporative latent heat) internal to freshly harvested peaches have a remarkable influence on its evaluation of cooling characteristics with respect to various cooling strategies. Therefore, to improve the accuracy of simulation results in peaches cooling, the term of heat source was coded as detailed procedures and included into a computational fluid dynamics (CFD) model. By comparing the temperature simulated with and without considering these heat sources, it is found that a reasonable decrease in variations of cooling performances is obtained with sustained increase in air‐inflow velocities. A maximum discrepancy in peaches volume‐weighted average temperature (∆*T*_vwa‐max_) is mainly concentrated in 0.1–0.3°C when the air‐inflow velocity not exceeds 1.7 m/s, and its corresponded 7/8ths cooling time (SECT) is also prolonged by 1–6 min. This means that, below 1.7 m/s, these heat sources should be added as a term into the heat transfer equations for modifying the mathematical model inside peaches computational domain. Furthermore, the feasibility of this modeling method is confirmed by a great agreement with experiments, and its modified model has a higher accuracy with the decreased RMSE and MAPE values of 6.90%–11.26% and 7.28%–12.95%, respectively.

## Graphical abstract

To improve the accuracy of simulation results in peaches cooling, these heat sources were coded as detailed procedures and included into a computational fluid dynamics (CFD) model. Beyond 1.7 m/s, the term of these heat sources can be ignored to save calculation cost. The feasibility of this modeling method is confirmed by a great agreement with experiments, and its modified model has a higher accuracy with the decreased RMSE and MAPE values of 6.90%–11.26% and 7.28%–12.95%, respectively.

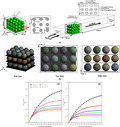

## 1 INTRODUCTION

A suitable low temperature environment, throughout the entire postharvest cold chain, is of great importance to maintain a high level of fruits and vegetables quality, and to extend their shelf life. Delays in cooling for over two hours promote quality deterioration and reduces their marketability, ascribing to the fact that the certain amount of field heat contained in freshly harvested fruits causes the fruits with high softening rate and respiration rate, especially for perishable commodities, such as peaches and strawberries. Therefore, the most crucial step for freshly harvested fruits is to remove field heat via prompt precooling, which retards after‐ripening and minimizes mass loss, prior to the refrigerated storage or transportation (Becker et al., 1996; Anderson et al., 2004). Forced‐air cooling (FAC), recommended for efficient precooling the horticultural fruits after harvest, is a typical industrial postharvest handling technique (Dincer, 1995; Brosnan & Sun, 2001).

In recent years, the application of computational fluid dynamics (CFD) in the research of postharvest precooling is becoming ever more popular. One of the key reasons is that the distributions of temperature and airflow can be obtained at a high spatio‐temporal resolution. Another reason is that the physical experimentation requires extensive human and material resources for field testing (Norton et al., 2007). Hence, CFD has been widely used to simulate the cooling phenomena of various horticultural food products, and then, the ventilation design of various cooling packing cartons has been optimized by comparison with precooling performances, such as cooling duration, mechanical strength, heterogeneity index, and convective heat transfer coefficient (Defraeye et al., 2013; Defraeye et al., 2015; Delele et al., 2013a; Delele et al., 2013b; Berry et al., 2016; O'Sullivan et al., 2017). In particular, the capability of CFD numerical models to predict the process of heat transfer, occurred inside individual cartons, was successfully confirmed. Because the accuracy of CFD simulation results was quantitatively validated against experimental date, the maximum root‐mean‐square error and standard error were mostly below 2°C, whereas the maximum mean absolute percentage error was <20% (Defraeye et al., 2013; Nalbandi et al., 2016; Han et al., 2015; Han et al., 2018). However, some developed models were idealized as plastic spheres instead of real fruits. Even few studies accounted for the effect of both respiratory and evaporative latent heat on heat flow inside the fruits zone, and most previous studies mainly considered the respiratory heat and neglected the importance of evaporative latent heat internal to fruit cooling (Dehghannya et al., 2011; Delele et al., 2008, 2013a; Delele et al., 2013b; Berry et al., 2016). Thus, the reliability of aerodynamic simulation results is reduced and the effect of evaporative cooling is not modeled explicitly. Han et al. (2017) found that the maximum temperature difference of a single apple was up to 0.033°C during cooling by comparing the temperature simulated with or without considering respiratory heat. Regarding strawberries, Nalbandi and Seiiedlou (2020) reported that transpiration heat could not be neglected during the cooling process, as the 7/8ths cooling time (SECT) decreased by 31% when this heat was added into the numerical model. Unfortunately, these previous researches did not analyze the sensitivities of these heat sources on various aspects of fruits cooling performances at different precooling strategies, resulting in an unclear relationship between these sensitivities and air‐inflow velocities, coupled with the uncertainty of its corresponding influence magnitude. Therefore, the calculation cost of simulating for fruit cooling cannot be adjusted according to different precooling strategies.

For peaches, most scholars mainly studied the effect of different cooling treatment on storage life and quality or studied its thermophysical parameters and perishability (Becker et al., 1996; Lurie & Crisosto, 2005; Yu et al., 2016; Zhou et al., 2019). Unfortunately, little researches have been conducted on whether respiration and transpiration heat generated inside the pulp of harvested peaches has a considerable effect on its variations of simulation results during the cooling process. However, peach is a perishable fruit, and an amount of heat sources will be generated from freshly harvested peach, which increases enzymatic activities, promotes microbial growth, accelerates its rot, and reduces the peaches commercial value of cold chain logistics. Hence, these heat sources cannot be ignored directly in simulating. To make the simulation process of heat and mass transfer more closer to the real experimental situation, an integrated computational procedure of the phenomena of respiration and evaporation in peaches zone was written in C programming language, and loaded into the computational domain of peaches by a user‐defined function (UDF).

The objective of this research was to develop a three‐dimensional CFD numerical model of a corrugated carton packed with multiple layers of peaches and to predict the process of forced‐convection cooling. More importantly, the developed mathematical model was modified by loading the respiration heat and evaporative latent heat into peaches computational domain for reducing the relative errors between experiment and simulation. Meanwhile, specific influence of these heat sources on peaches precooling properties variations (including the cooling rate and uniformity) was also sensitively analyzed to adjust the calculation cost of peaches numerical simulation on different air‐inflow velocities. Furthermore, the usability and accuracy of this modeling method are also needed to be further verified through experiments.

## 2 MATERIALS AND METHODS

### 2.1 Physical model

**FIGURE 1:**
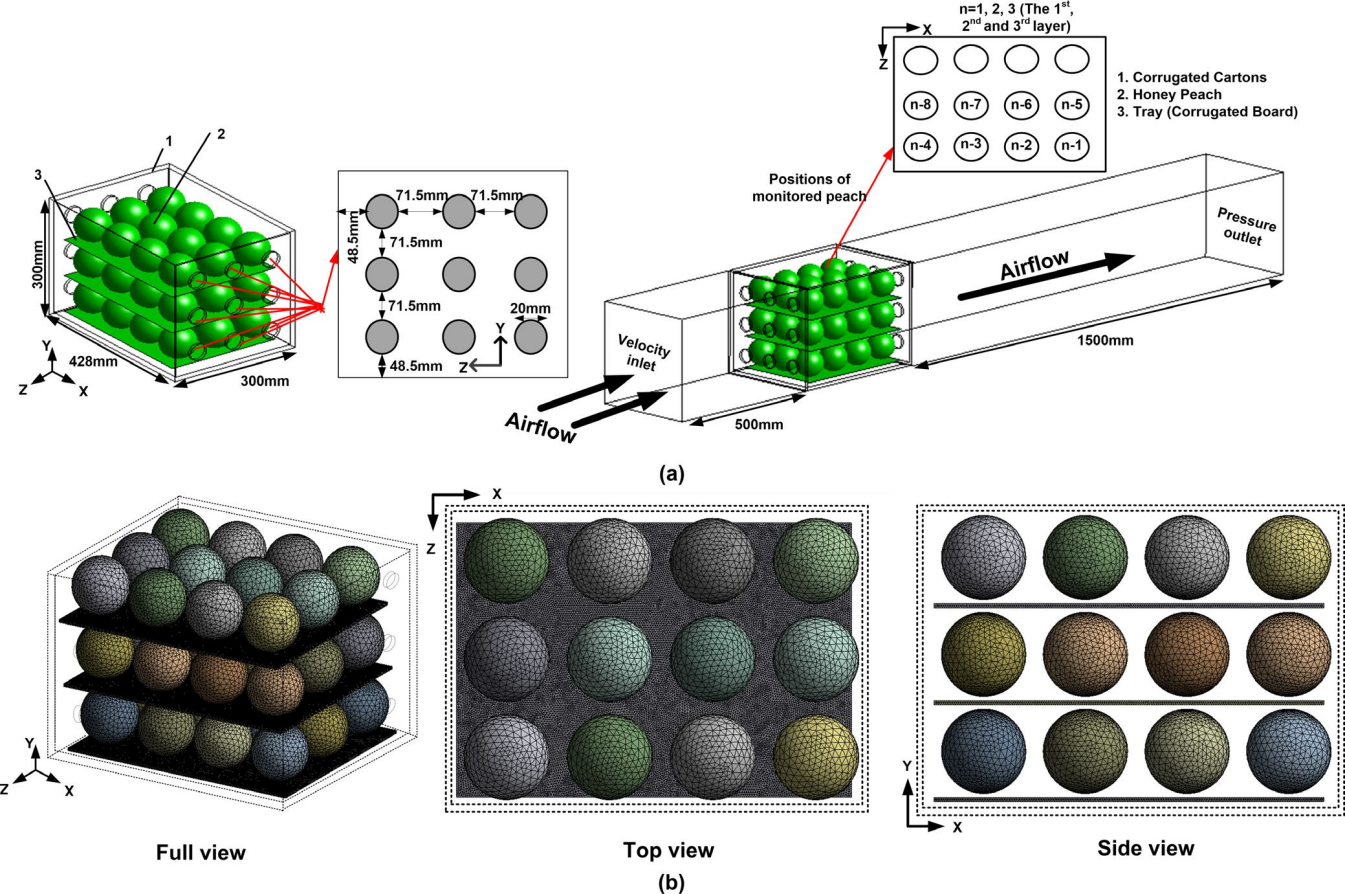
(a) Schematic diagram of precooling simulation: positions of monitored peaches inside an individual packages (from *n* − 1 to *n* − 8, where *n* = 1, 2, 3), (b) computational grids of numerical model

To make peaches cool more evenly and rapidly in vented packaging cartons, the ventilation parameters were adjusted reasonably by analyzing the characteristics of its shape and size. Meanwhile, the total opening area (TOA) of short side for this corrugated carton was designed as 3.14%, which satisfied the structure requirement of commodity packages. Because the majority of telescopic containers, used for export, had an average ventilated area percentage of 4% (Berry et al., 2016), a more uniform airflow distribution was produced by increasing the vent area from 1% to 7%, which minimized compression strength loss and much larger increased in cooling rate (Delele et al., 2013a; Delele et al., 2013b). We used Design Modeler of ANSYS19.2 (ANSYS Inc.) to develop this carton with symmetrical vents, which were made of double layer reinforced corrugated carton of 428 × 300 × 300 mm^3^, and its thickness was 7 mm. The tray was a single corrugated cardboard of 368 × 256 × 4 mm^3^. The geometrical dimensions of cartons and its precooling simulation settings are shown in Figure 1a.

### 2.2 Meshing sensitivity

A critical step after designing system geometry was to discretize it into a three‐dimensional computational mesh (see Figure 1b). The preprocessor software of Meshing was adopted to discretize the computational domain into a tetrahedral hybrid grid. According to the study of mesh sensitivity, meshes with a maximum edge length of 5 mm in peaches and 1 mm in other regions were found to be the optimum sizes, which was accorded with O'Sullivan et al. (2016) recommendation that the maximum size on any individual face was limited to 7.5 mm. Additionally, the growth rate between cells was remained at 1.2, lower than the maximum value of 1.3 proposed by Franke et al. (2007). Finally, through the examination of meshes quality, it was found that the maximum skewness and the calculated wall *y*+ values were respectively lower than 0.85 and 6.0, which is in line with the detection of CFD grid quality proposed by previous scholars (Dehghannya et al., 2008; Han et al., 2015; Han et al., 2017; Delele et al., 2013a; Delele et al., 2013b), that is, the skewness for tetrahedral meshes of models should be controlled within 0.95. Thus, a high and rational mesh quality of this model was successfully verified by this evidence, thereby further ensuring the feasibility of fluid‐structure coupling in this numerical model.

The model was discretized into an unstructured grid of 6,964,947 elements. Smaller meshes than that size presented the same simulation results (i.e., wall *y*+ value as well as skewness and peaches temperature), but increased the calculation cost. Individual peaches were modeled discretely as spheres with a diameter of 80 mm. To facilitate grid division, a certain space should be left between containers and peaches, trays and peaches, and peaches and peaches (Tutar et al., 2009).

### 2.3 Numerical solution procedure

To simulate the dynamics, we used a transient simulation with a time step of 20 s and 20 iterations per time step. The accuracy of CFD simulation results, based on Reynolds averaged Navier Stokes (RANS), depends to a large extent on turbulence models and boundary‐layer modeling method used (Berry et al., 2016). The surfaces of corrugated cartons and peaches were defined as a no slip wall with zero roughness and the shear stress transport (SST) k‐w model was adopted in this work (Norton et al., 2007; Defraeye et al., 2012).

With a used pressure‐based split solver, the discrete format of momentum, energy, turbulent kinetic energy, and diffusivity was all set to second‐order upwind scheme. The semi‐implicit method for pressure‐linked equations (SIMPLE) was utilized to couple pressure to velocity. The convergence criterion of continuity, momentum, and turbulence was 10^−4^, and that of energy equation was 10^−6^. Before simulating, the initial and boundary conditions of this numerical model should be defined (see Figure 1a). The inlet of the computational domain was set as a velocity‐inlet boundary condition, performed by using six different air‐inflow velocities: 0.2, 0.7, 1.2, 1.7, 2.2, 2.7 m/s. The outlet of the computational domain was set as a pressure‐outflow boundary condition, and its under pressure outlet was caused by the fan in a forced‐air tunnel cooler device. The initial temperature of peaches and the precooling‐air temperature in refrigerated tunnel were respectively set to 26 and 2°C. The thermal‐physical parameters of each object in this model are shown in Table 1. The simulation was run on a 64‐bit windows10 computer with a 2.90 GHZ InterCore TM i7‐7500U CPU and 8GB RAM as well as a 64‐bit windows10 computer with a 2.30 GHZ Inter Core E5‐2697 V4 CPU and 256GB RAM.

**TABLE 1:** Parameters of thermal‐physical properties

Parameters	Density (kg/m^3^)	Specific heat capacity (J kg^−1^ K^−1^)	Thermal conductivity (W m^−1^ K^−1^)	Dynamic viscosity (Pa s)
Precooling‐air	1.293	1006	0.02343	1.73e−5
Peach	691.95	3898.3	0.472	—
Corrugated Carton	195	1700	0.065	—
Tray (Corrugated Board)	195	1700	0.065	—

### 2.4 Mathematical modeling

A commercial CFD code of ANSYS 19.2 was used to develop a 3‐D model of the horticultural product packaging system and stacking. The computational domain of this model can be divided into three distinct sub‐domains: free air flow zone, the solid zone of corrugated container, and solid peaches zone.

#### 2.4.1 Governing equations

The Reynolds‐average Navier–Stokes equations are utilized to solve the air flow (an incompressible fluid) in the region of free airflow.

Mass conservation equation

(1) $$\frac{\partial \rho_{a}}{\partial t}+\text{div}\left(\rho_{a}U\right)=0$$

Momentum conservation equation

(2a) $$\frac{\partial (\rho_{a}u)}{\partial t}+\text{div}\left(\rho_{a}uU\right)=\text{div}\left(\mu_{a}\text{grad}u\right)‐\frac{\partial P_{a}}{\partial x}+\left[‐\frac{\partial (\rho_{a}\bar{u^{\prime 2}})}{\partial x}‐\frac{\partial (\rho_{a}\bar{u^{\prime }v^{\prime }})}{\partial y}‐\frac{\partial (\rho_{a}\bar{u^{\prime }w^{\prime }})}{\partial z}\right]+S_{u}$$

(2b) $$\frac{\partial (\rho_{a}v)}{\partial t}+\text{div}\left(\rho_{a}vU\right)=\text{div}\left(\mu_{a}\text{grad}v\right)‐\frac{\partial P_{a}}{\partial y}+\left[‐\frac{\partial (\rho_{a}\bar{v^{\prime 2}})}{\partial y}‐\frac{\partial (\rho_{a}\bar{v^{\prime }w^{\prime }})}{\partial z}‐\frac{\partial (\rho_{a}\bar{u^{\prime }v^{\prime }})}{\partial x}\right]+S_{v}$$

(2c) $$\frac{\partial (\rho_{a}w)}{\partial t}+\text{div}\left(\rho_{a}wU\right)=\text{div}\left(\mu_{a}\text{grad}w\right)‐\frac{\partial P_{a}}{\partial z}+\left[‐\frac{\partial (\rho_{a}\bar{w^{\prime 2}})}{\partial z}‐\frac{\partial (\rho_{a}\bar{u^{\prime }w^{\prime }})}{\partial x}‐\frac{\partial (\rho_{a}\bar{v^{\prime }w^{\prime }})}{\partial y}\right]+S_{w}$$

Energy conservation equation

(3) $$\frac{\partial T_{a}}{\partial t}+\text{div}\left(UT_{a}\right)=\text{div}\left(\frac{\lambda_{a}}{\rho_{a}c_{a}}\text{grad}T_{a}\right)‐\frac{\partial (\bar{u^{\prime }T_{a}^{\prime }})}{\partial x}‐\frac{\partial (\bar{v^{\prime }T_{a}^{\prime }})}{\partial y}‐\frac{\partial (\bar{w^{\prime }T_{a}^{\prime }})}{\partial z}$$

where *c_a_* and *ρ_a_* are the specific heat capacity (J kg^−1^ K^−1^) and density of air (kg/m^3^), respectively. *U* and *P_a_* are the air velocity (m/s) and water vapor pressure inside box (Pa), among them, *u*, *v*, *w* mean the velocity component in the *X*, *Y*, *Z* directions, respectively. *µ_a_* and *λ_a_* represent the dynamic viscosity (Pa s) and thermal conductivity of air (W m^−1^ K^−1^), respectively. *T_a_* and *t* denote the air temperature (K) and cooling time (s), respectively. The effect of gravity in free airflow zone is only taken into consideration in this study, thereby *S_u_* = *S_w_* = 0, *S_v_* = −*ρ_a_g*, where *S_u_*, *S_v_*, *S_w_* indicate a source term in the *X*, *Y*, and *Z* directions, and *g* represents the acceleration (9.81 m/s^2^) due to gravity.

#### 2.4.2 Modifying the developed mathematical model inside peaches computational domain

Heat transfer in the peaches domain was modeled using Equation 4 with internal heat sources (*Q*_int_, W/m^3^), including respiratory heat (*Q_r_*, W/m^3^) and evaporative latent heat (*Q_e_*, W/m^3^).

(4) $$\lambda_{p}\left(\frac{\partial^{2}T_{p,t}}{\partial r^{2}}+\frac{2}{r}\frac{\partial T_{p,t}}{\partial r}+\frac{\cos\theta }{r^{2}\sin\theta }\cdot \frac{\partial T_{p,t}}{\partial \theta }+\frac{1}{r^{2}}\frac{\partial^{2}T_{p,t}}{\partial \theta^{2}}\right)+Q_{\text{int}}=c_{p}\rho_{p}\frac{\partial T_{p,t}}{\partial t}$$

where *c_p_* and *ρ_p_* are the specific heat capacity (J kg^−1^ K^−1^) and density of peaches (kg/m^3^), respectively. *λ_p_* is peaches thermal conductivity (W m^−1^ K^−1^). While *V_p_* and *A_p_* represent peaches volume (m^3^) and surface area (m^2^), respectively, and *r* is its vector radius (m). *T_p_*_,_ *_t_* is peaches temperature at time *t* (K).

Many developed mathematical models in previous studies have neglected the heat sources internal to the produce, that is, *Q*_int_ = 0, or coded only respiratory heat in simulating. However, for extremely perishable fruits, the model should be modified by considering both respiration heat and evaporative latent heat, generated internal to the pulp of peaches, to reduce the error between experiment and simulation. Its detailed calculation formulas are as follows:

(5) $$Q_{\text{int}}=\left(Q_{r}‐Q_{e}\right)/V_{P}$$

(6) $$Q_{r}/V_{p}=\rho_{p}\times f_{p}$$

(7) $$Q_{e}/V_{p}=L_{p}m_{p}A_{p}/V_{p}=3L_{p}m_{p}/r$$

In these equations, *f_p_* is the respiratory heat generation per unit mass of commodity (W/kg), and $f_{p}=\left(10.7/3600\right)\times A\times {\left[1.8\left(T_{p,t}‐273.15\right)+32\right]}^{B}$, where the respiration coefficients (A and B) are given in Becker et al. (1996) and successfully applied in many previous researches (Delele et al. 2013a; Han et al. 2015; Han et al., 2017), for peaches these values are 1.2996 × 10^−5^ and 3.6417, respectively. *L_p_* represents the latent heat of evaporation (J/kg), $L_{p}=9.1T_{p,t}^{2}‐7,512.9T_{p,t}+3,875,100$. *m_p_* is the rate of moisture loss from fruits (kg m^−2^ s^−1^), which is estimated as follows (Hoang et al., 2003):

(8) $$m_{p}=k_{\text{ps}}\left(P_{\text{ps}}‐P_{a}\right)$$

where *P*_ps_ is the water vapor pressure surrounding the fruits surface, and *P_a_* is the water vapor pressure in air (Pa), *P*_ps_ = VPL × *P_w_*(*T_a_*) and *P_a_* = RH × *P_w_*(*T_a_*) (Han et al., 2015; Han et al., 2017; Zhao et al., 2016), and *T_a_* is the cold air temperature inside the refrigerated system (K) with a relative humidity of cold air RH = 90%. VPL is the vapor pressure lowering effect of various fruits and vegetables given by Becker et al. (1996), for peaches, VPL = 0.99.

The quantity *P_w_* denotes the saturation partial water vapor pressure (Pa), which can be calculated from the Antoine equation (Ferrua and Singh, 2009a).

(9) $$P_{W}=\exp\left(23.4795‐\frac{3990.5}{T‐39.317}\right)$$

The mass transfer coefficient (*k*_ps_, kg m^−2^ s^−1^ Pa^−1^) is modeled as (Dehghannya et al., 2008):

(10) $$k_{\text{ps}}=\frac{1}{1/k_{a}+1/k_{s}}$$

where *k_s_* and *k_a_* are the skin mass transfer coefficient and air film mass transfer coefficient, respectively, among them, *k_s_* for various fruits and vegetables is tabulated by Becker et al. (1996), for peaches, *k_s_* = 14.2 × 10^−9^ (kg m^−2^ s^−1^ Pa^−1^). The value of *k_a_* is obtained through the calculation on the mass transfer correlation Sherwood‐Reynolds‐Schmidt (Han et al., 2017):

(11) $$Sh=\frac{k_{a}\cdot 2r\cdot R_{H_{2}O}\cdot T_{a}}{\delta M_{H_{2}O}}=2\text{.0}+0\text{.552}Re^{0\text{.53}}Sc^{0\text{.33}}$$

(12) $$\delta =\frac{9.1\times 10^{‐9}\times T_{a}^{2.5}}{T_{a}+245.18}$$

Assuming negligible airflow around peaches, that is *Re* ≈ 0, and the mass transfer coefficient of air film can be expressed by following equation (Rennie and Tavoularis, 2009):

(13) $$k_{a}=\delta M_{H_{2}O}/(R_{H_{2}O}T_{a}r)$$

where *M*_H2O_ = 0.018 is the molecular mass of water vapor (kg/mol), *R*_H2O_ = 8.314 is the water vapor constant (J mol^−1^ K^−1^). *δ* = 2.196 × 10^−5^ is the diffusion coefficient of water vapor in air (m^2^/s) when *T_a_* = 275.15 K. At this point, the value of *k*_ps_ and *k_a_* can be estimated as 3.313 × 10^−9^ and 4.320 × 10^−9^ (kg m^−2^ s^−1^ Pa^−1^), respectively.

### 2.5 Evaluation of precooling performances

#### 2.5.1 Cooling rate and SECT

The cooling rate is frequently evaluated by adopting dimensionless temperature (*Y*), which is determined from the temperature‐time profile of the internal product temperature (*T_p_*_,_ *_t_*) (Dincer 1995; Defraeye et al., 2015).

(14) $$Y_{t}=\frac{T_{p,t}‐T_{a}}{T_{p,0}‐T_{a}}$$

(15) $$T_{p\text{, vwa}}=\frac{1}{V_{P}}\sum_{i=1}^{N}V_{i}T_{i}\cdot T_{p\text{, swa}}=\frac{1}{A_{P}}\sum_{i=1}^{N}A_{i}T_{i}$$

(16) $$\Delta T_{t}=T_{\text{with}‐\text{heat},t}‐T_{\text{without}‐\text{heat},t}$$

where *T_p_*_,0_ is the initial peaches temperature, *T_p_*_,vwa_ and *T_p_*_,swa_ (°C) are the volume and surface area‐weighted average temperature of peaches, respectively. *T_i_* (°C) is the temperature at cell position *i* = 1 to *i* = *N*, and *V_i_* (m^3^) and *A_i_* (m^2^) are the volume and area of mesh cell *i*. ∆*T_t_* (°C) is the temperature variation of the peach in cooling by comparing the temperature simulated with (*T*_with‐heat,_ *_t_*) and without (*T*_without‐heat,_ *_t_*) internal heat sources (*Q*_int_).

For the precooling process, the 7/8ths cooling time (SECT, min, *Y* = 0.125) is generally used for the commercial forced‐air precooling operation, because from then on, the temperature of product is acceptably close to the required storage temperature. Then, products can be transferred to the refrigeration equipment where the remaining heat load can be eliminated with less energy consumption. Hence, SECT is considered a robust parameter for comparing cooling rates (Brosnan and Sun, 2001).

#### 2.5.2 Cooling uniformity

Olatunji et al. (2017) indicated a new heterogeneity index (HI), which quantified the levels of cooling uniformity over the entire process time from a product‐side perspective, namely, the overall heterogeneity index OHI = ∆*Y*_max_ − ∆*Y*_min_. A lower value of OHI presents better homogeneity over the whole processing time, conversely, the worse uniformity of temperature distribution is.

(17) $$Y_{\text{avg},t}=\sum_{n=1}^{m}Y_{n,t}/m$$

(18) $$\Delta Y_{n,t}=Y_{n,t}‐Y_{\text{avg},t}$$

(19) $${\text{HI}}_{t}=\Delta Y_{\max‐P,t}‐\Delta Y_{\min‐N,t}$$

where *Y*_avg,_ *_t_* is the average dimensionless temperature of all monitored fruits, ∆*Y*_max‐_ *_P_*_,_ *_t_* and ∆*Y*_min‐_ *_N_*_,_ *_t_* are the maximum and minimum values of ∆*Y_n_* at single time points, HI*_t_* is the instantaneous cooling homogeneity at a certain moment.

### 2.6 Validating the model with experiments

To determine the validity of this mathematical model, predicted (*S_i_*) and measured (*E_i_*) temperature are compared based on the root‐mean‐square error (RMSE) and mean absolute percentage error (MAPE).

(20) $$\text{RMSE}=\sqrt{\frac{1}{n}\sum_{i=1}^{n}{\left(E_{i}‐S_{i}\right)}^{2}}$$

(21) $$\text{MAPE}=\frac{1}{n}\sum_{i=1}^{n}\frac{\left|E_{i}‐S_{i}\right|}{E_{i}}\times 100\%$$

**FIGURE 2:**
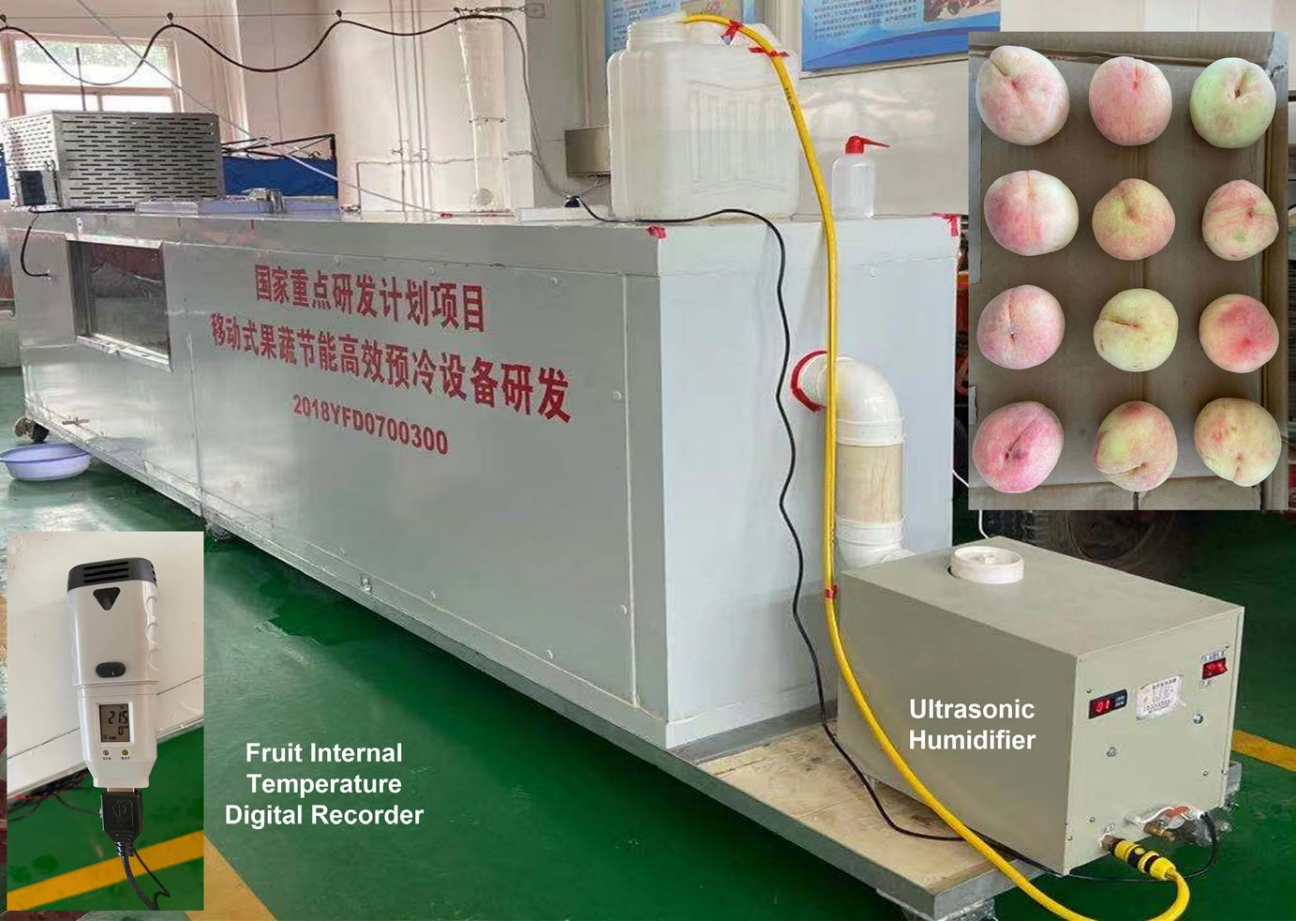
Forced‐air cooling system and the stacking position of peaches in experiments

Fresh okubo peaches were picked in July from Taigu of Shanxi Province (112°55′E, 37°43′N) and then cooled immediately by using a self‐made forced‐air cooling system (see Figure 2) with temperature and relative humidity of 2°C and 80%–90%, respectively. The fruit temperature was measured with a temperature digital recorder (SSN‐13E, YOWEXA, Inc.). An accuracy of this sensor is ±0.3°C. The fog making capacity and power consumption of ultrasonic humidifier (HS‐05‐3, LUOSHE HUASHENG, Inc.) are 0.3 and 0.2 KW, respectively.

## 3 RESULTS AND DISCUSSION

### 3.1 Effect of internal heat sources on variations of temperature and cooling time

**FIGURE 3:**
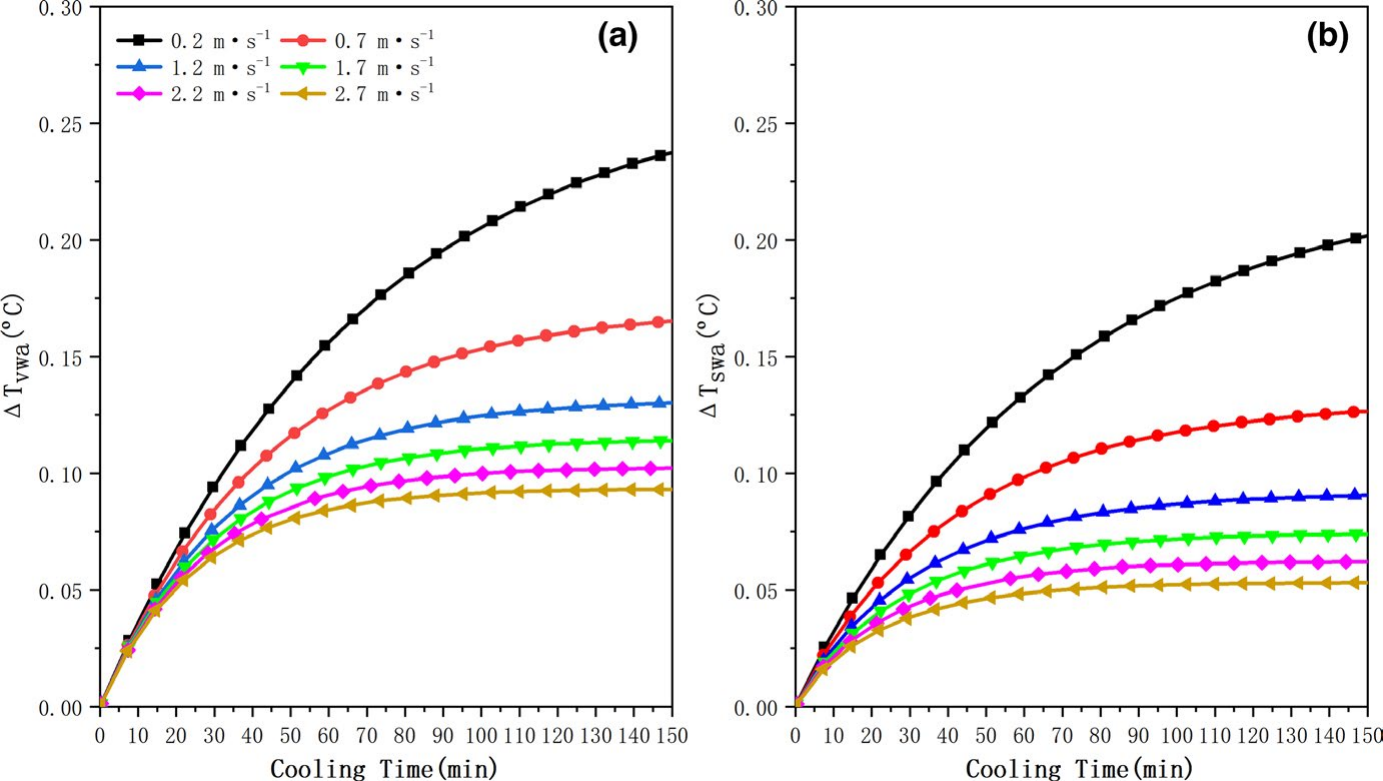
Temperature variations (∆*T*) as a function of cooling time: monitored position at peach 1‐1

CFD numerical model predicted the complex temperature distribution at a given moment with and without considering the internal heat sources, as expressed by Equation 16. Figure 3 illustrates the curves of variations in ∆*T* versus cooling time, where ∆*T*_vwa_ and ∆*T*_swa_ are the variations of volume and surface area‐weighted average temperature, respectively. As can be observed intuitively from Figure 3a, there is an increase in peaches temperature within the cooling time of 150 min (i.e., ∆*T*_vwa_ > 0), compared with the simulated temperature without coding heat sources internal (*Q*_int_ = *Q_r_* − *Q_e_*) into its computational domain. In particular, the value of ∆*T*_vwa_ is significantly increased during its corresponding 7/8ths cooling time (SECT) of various precooling conditions, because heat source internal to the peach is always in the state of exothermic during the 7/8ths cooling time (i.e., *Q*_int_ > 0), even if the internal heat source decreases with the increasing of cooling time. This means that, in this period, the total amount of evaporative latent heat (*Q_e_*) internal to the pulp of peaches is slightly lower than that of respiration heat (*Q_r_*), which also can be obtained through statistics (coded by Equations (5), (6), (7), (8), (9), (10), (11), (12), (13)). Simultaneously, this is also the reason why the SECT is prolonged, which is discussed in the following section. These internal heat sources are then absorbed by the phenomenon of convective heat transfer between fruits and precooling air.

The curve of ∆*T*_swa_ follows similar trend to ∆*T*_vwa_ (see Figure 3b), but the value of ∆*T*_swa_ is always lower than that of ∆*T*_vwa_. This result is mainly attributed to the effect of respiratory heat on the flesh more than on the epidermis of fruits. As expected from the data analysis in Table 2, during the entire forced‐convection cooling, the maximum temperature differences of an individual peaches (∆*T*_vwa‐max_) and of peaches surface (∆*T*_swa‐max_) are as high as 0.258°C and 0.219°C, respectively. Meanwhile, the temperature values simulated with or without heat sources are compared by linear regression method. When air‐inflow velocity is equal to 0.2 m/s (*V*_inlet_ = 0.2 m/s), the regression equation has an intercept (*b*) of 0.28849 and a slope (*k*) of 0.98907, and its adjusted correlation coefficient (*R* ^2^) is 1. Then, the intercept (*b*) and temperature variations (∆*T*_vwa_ and ∆*T*_swa_) are respectively decreased with the increase of air‐inflow velocities, owing to the fact that internal heat source decreases with a continuous increasing of air‐inflow velocity, among them, air‐inflow velocity is more sensitive to the magnitude of decreased respiration heat than that of increased evaporative latent heat internal to fruits. Particularly when an air‐inflow velocity exceeding 1.7 m/s, the value of ∆*T*_vwa_ changes slowly and the ∆*T*_vwa‐max_ starts to below ~0.1°C.

**TABLE 2:** Linear regression function between simulated volume‐weighted average temperature with and without accounting for heat sources inside peaches zone (1‐1), and its corresponded 7/8ths cooling time (SECT, s)

Air‐inflow velocity (m/s)	*T*_vwa‐with_ = *k *· *T*_vwa‐without_ + *b*			Δ*T*_max_ (°C)		Whole		Surface		
	*k*	*b*	*R* ^2^	Δ*T*_vwa‐max_	Δ*T*_swa‐max_	SECT_without_	SECT_with_	SECT_without_	SECT_with_	
0.2	0.98907	0.28849	1	0.258	0.219	8520	8860	7800		8080
0.7	0.99287	0.18533	1	0.170	0.130	5500	5620	4700		4800
1.2	0.99441	0.14319	1	0.132	0.092	4260	4360	3400		3460
1.7	0.99509	0.12451	1	0.115	0.075	3740	3800	2820		2860
2.2	0.99557	0.11137	1	0.103	0.062	3360	3400	2400		2420
2.7	0.99592	0.10153	1	0.094	0.053	3080	3120	2060		2080

When *V*_inlet_ = 2.7 m/s, the ∆*T*_vwa‐max_ of a single peach is 0.094°C, which is higher than the simulation results of Han et al. (2017) who found that the ∆*T*_vwa‐max_ of an apple between the temperature simulated with and without accounting for respiratory heat was ~0.033°C when *V*_inlet_ = 2.5 m/s. This is mainly due to the fact that the breathing effect of peaches is more vigorously than apples, which makes them more likely to rot and lose commercial edible value. For example, respiratory heat of a peach is about twofold that of an apple when the initial temperature of both fruits is set to 27°C, calculated by correlating Equation 6, among them, for apples, *A* = 5.6871 × 10^−4^ and *B* = 2.5977 (Becker et al., 1996).

By comparing the differences of SECT (∆SECT) between the results simulated with and without internal heat sources, it is found that, with respect to various precooling strategies, the SECT is also prolonged by 340, 120, 100, 60, 40, and 40 s, respectively. This result indicates that, during the 7/8ths cooling time, an additional of heat sources term in the mathematical model of peaches computational domain affects cooling rate, resulting from the increased heat transfer resistance which is caused by respiratory heat internal to fruits. Additionally, this reflects the inverse relationship between the ∆SECT and air‐inflow velocity. However, when an air‐inflow velocity exceeding 1.7 m/s, the ∆SECT of various precooling conditions starts to be lower than 1 min and decreases slowly by increasing air‐inflow velocities. Whereas for peaches surfaces, there is also existing an explicit variation in SECT when *V*_inlet_ = 0.2 m/s, that is, an increase of 3.59% (4.67 min). Based on these observations, when *V*_inlet_ < 1.7 m/s, the heat generation by the pulp of peach during the cooling process has a significant influence not only on temperature distribution but also on SECT.

### 3.2 Effect of internal heat sources on variations of cooling uniformity

**FIGURE 4:**
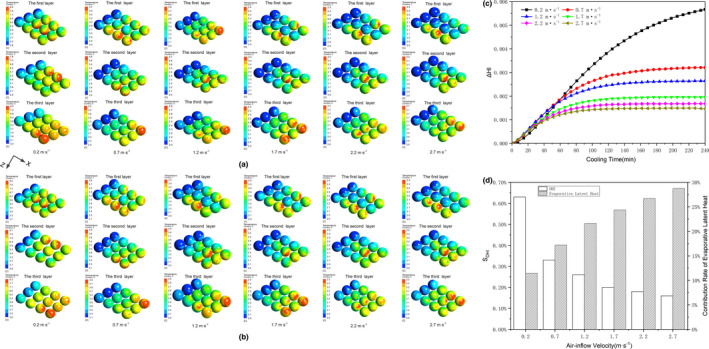
Instantaneous static temperature (°C) contours around each layer (1st, 2nd, and 3rd layer) of peaches for different air‐inflow velocities at the simulation time of 120 min: the CFD model is simulated (a) without and (b) with internal heat sources; (c) Comparison of heterogeneity index (HI) between the temperature simulated with and without heat source: ∆HI = HI_with_ − HI_without_; (d) The sensitivity of overall heterogeneity index as a function of air‐inflow velocities: SOHI = (OHI_with_ − OHI_without_)/OHI_without_, and contribution rate of evaporative latent heat from a single peach 1‐4 during 4 hr of precooling

By comparing the differences of cooling uniformity at various working conditions (see Figure 4c), we find that adding heat source term to the mathematical models has a negative effect on instantaneous cooling uniformity (i.e., HI_with_ > HI_without_). This is ascribed to an increase in temperature gradient from entrance to exit vents (see Figure 4a,b). In particular, a difference of temperature gradient between these two cooling conditions is as high as 0.2°C when *V*_inlet_ = 0.2 m/s, simultaneously, the sensitivity of OHI (S_OHI_) is 0.63% within the precooling time of 240 min. However, the *S*_OHI_ shows a slow decreasing trend when the air‐inflow velocity exceeds 1.7 m/s, that is, S_OHI_ < 0.2% and ∆HI < 0.002 (see Figure 4c,d).

These analyses are clearly demonstrated that, when air‐inflow velocity increases to more than 1.7 m/s, modifying the developed mathematical model with an additional of these heat sources has almost no considerable influence on quantifying the variations of precooling performances. This is mainly because air‐inflow velocity is no longer a major factor that significantly affects the cooling capability of peach when the air‐inflow velocity exceeds 1.7 m/s, observed from Table 2, Figure 4a or b. When the term of heat source is loaded into mathematical model, the variations of SECT and temperature distribution for different air‐inflow velocities are lower than 8 min and 0.3°C, respectively. So does the CFD model simulated without internal heat sources.

### 3.3 Importance of moisture loss term in cooling process

To further investigate the importance of moisture evaporation in peaches cooling, the ratio between the evaporative latent heat of peaches and its total amount of heat lost by convective heat transfer is estimated. During the 4 hr of precooling, the moisture loss is assessed at 0.15% (RH = 90%, *V*_inlet_ = 0.2 m/s), and the contribution rate of evaporative latent heat is increased from 11.46 to 28.78% with an increasing of air‐inflow velocity, observed from Figure 4d. Besides, when the strawberries were precooled for 2 hr with dry air, the moisture loss for each individual clamshell was estimated at 0.3%–0.54%, and the average contribution of moisture loss was estimated to be between 15% and 26% (Ferrua and Singh, 2009b). A moisture loss of waxed citrus fruits was 0.42% during the cooling period of 17.6 hr that corresponded to an evaporative cooling of 12.79% (Lambrecht, 2012). Thus, the moisture loss term inside fruits should be included in heat sources term during the cooling process, which is consistent with the investigated results of Nalbandi and Seiiedlou (2020). This is due to the fact that the associated error can be compensated by using the mathematical coupling of the term of moisture loss to the airflow.

### 3.4 Experimental verification

**FIGURE 5:**
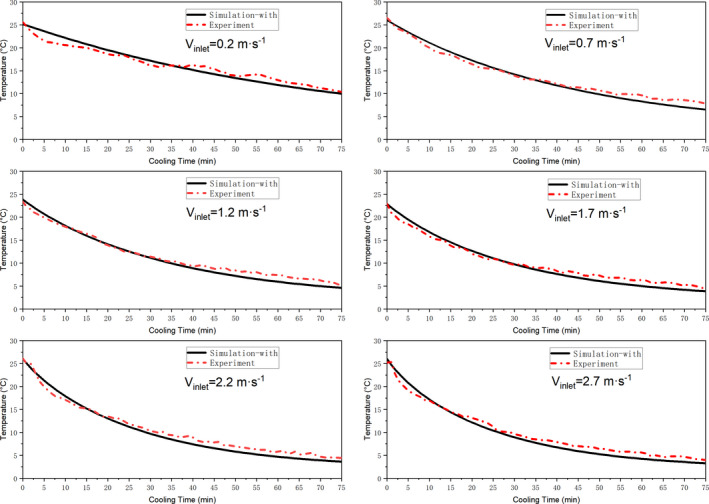
Simulated (with heat source) and experimental temperature profiles based on different air‐inflow velocities during the 75 min cooling: monitored position at peach 1‐1

Figure 5 compares the simulated (with heat sources) and experimentally obtained temperature as function of cooling time for six different air‐inflow velocities (0.2–2.7 m/s). Overall, the simulated temperatures are basically in good agreement with the measured results. Compared with the results simulated without considering the term of internal heat sources (its corresponding values of RMSE and MAPE are concentrated in 0.817–0.918°C and 4.40%–11.54%), the modified numerical model has a higher accuracy with RMSE and MAPE values in the range of 0.725–0.852°C and 3.83%–10.70%, respectively (see Table 3). As a result, the RMSE values of the modified numerical model for various air‐inflow velocities are decreased of 11.26%, 9.89%, 8.70%, 8.61%, 7.19%, 6.90%, respectively, whereas the MAPE values are also decreased of 12.95%, 10.23%, 9.39%, 9.00%, 7.74%, 7.28%, respectively. Based on the obtained results, the mathematical model with the term of internal heat sources and 3D simulator can more accurately simulate the precooling process of peaches. More significantly, the improvement rate of its accuracy basically shows a downward trend as the air‐inflow velocity increases, especially when *V*_inlet_ > 1.7 m/s, the reduction rates of RMSE and MAPE are both <8.00%, which has further validated the conclusion of the previous discussion.

**TABLE 3:** Validation results between the numerical model (with or without considering internal heat source) and experiment at various conditions

Air‐inflow Velocities (m/s)	Simulation‐without internal heat sources						Simulation‐with internal heat sources					
	0.2	0.7	1.2	1.7	2.2	2.7	0.2	0.7	1.2	1.7	2.2	2.7
RMSE (°C)	0.817	0.829	0.897	0.836	0.918	0.898	0.725	0.747	0.819	0.764	0.852	0.836
MAPE (%)	4.40	6.06	8.73	9.56	10.73	11.54	3.83	5.44	7.91	8.70	9.90	10.70

## 4 CONCLUSIONS

This research established a three‐dimensional mathematical model (with the term of heat sources) of airflow and heat transfer for analyzing the aerodynamic and forced‐convection cooling process inside individual corrugated cartons. The influence of considering heat sources on various cooling performances was also comprehensively investigated and compared. The results indicate that adding the term of these heat sources in the equations presents a significant effect on these variations of cooling performances when the air‐inflow velocity not exceeds 1.7 m/s. However, any further increase in air‐inflow velocity will lead to a relative low decrease in the variations of overall heterogeneity index (OHI) and 7/8ths cooling time (SECT), which are lower than 0.2% and 1 min, respectively, and the effect of temperature will begin to below ~0.1°C after this point. Therefore, beyond 1.7 m/s, the term of these heat sources can be ignored to save calculation cost. Meanwhile, experimental results have successfully verified the feasibility and accuracy of this modified model with the decreased RMSE and MAPE values of 6.90%–11.26% and 7.28%–12.95%, respectively. This study provides a reliable theoretical reference for reducing the relative error of experimental and simulated results, and further adjusting the numerical calculation cost according to different precooling strategies.

## CONFLICT OF INTEREST

Authors declare that they do not have conflict of interest.

## ETHICAL APPROVAL

This study does not involve any human or animal testing.
